# Supplementary material for: An electron counting algorithm improves imaging of proteins with low-acceleration-voltage cryo-electron microscope
Source: Commun Biol. 2022 Apr 6;5:321. doi: 10.1038/s42003-022-03284-1 (PMC8987035; doi:10.1038/s42003-022-03284-1)
Supplement: Supplementary file 2 — Supplementary Information [file 42003_2022_3284_MOESM2_ESM.pdf]

An electron counting algorithm improves imaging of proteins with low-acceleration-voltage cryo-electron microscope

Zhu et al.

Supplementary Information

Supplementary Figure 1

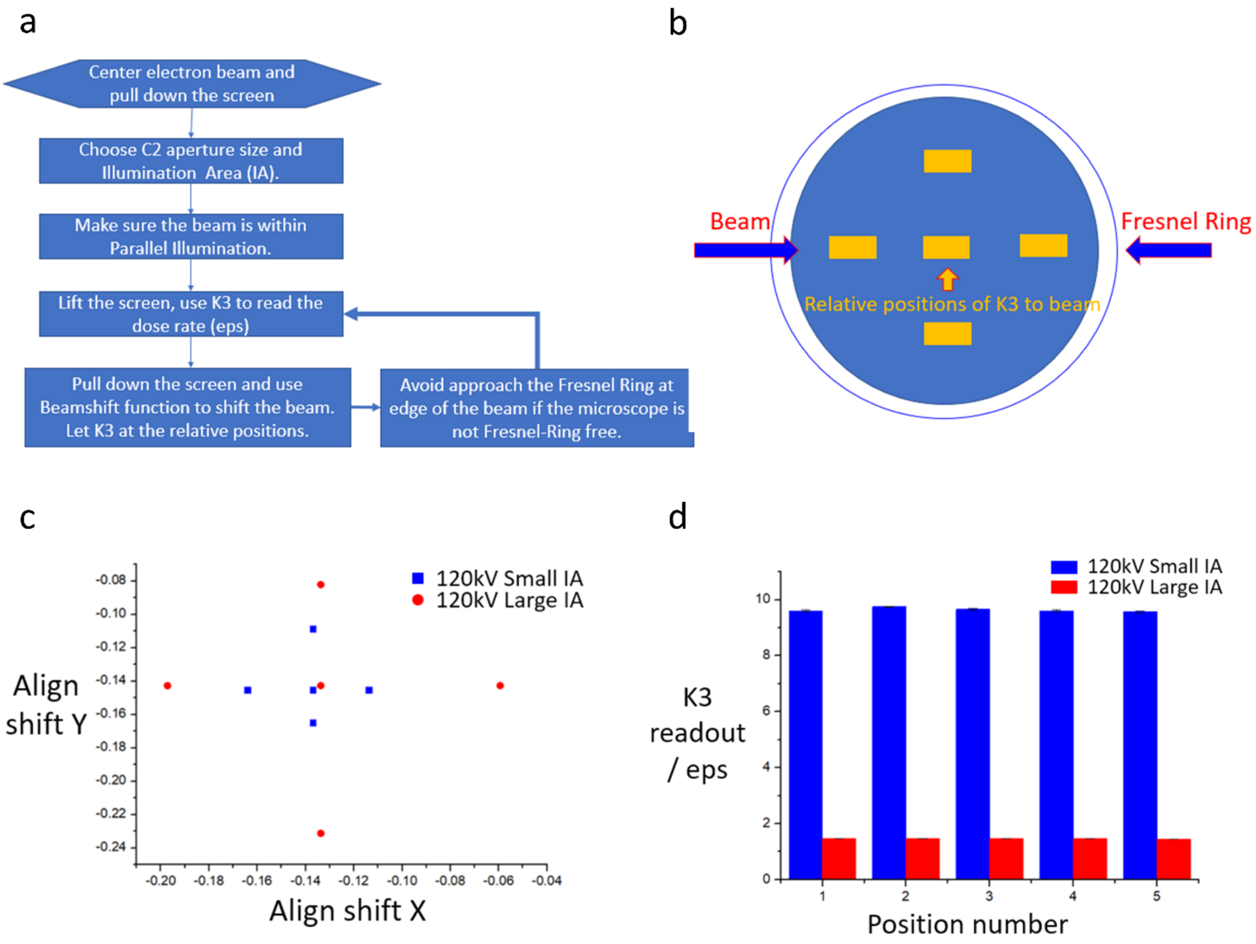

Supplementary Figure 1. Workflow and results of electron beam homogeneity measurements.

a. The workflow chart for measuring homogeneity of electron beam. b. Schematic of the five positions of K3 camera related to beam. The orange squares represent K3, the solid blue circle represents the beam and outer ring represents the Fresnel Ring of the beam. c. Actual positions of beam-centre with “align shift X/Y”. Blue squares are for small illuminated area (IA) (high intensity) and red dots are for large illuminated area (low intensity). d. The readouts of K3 in small/large illuminated area. Error bars representing 1 standard deviation were marked on the graph. The standard error of small IA is 0.69% and 0.66% for large IA at 120 kV.

Supplementary Figure 2

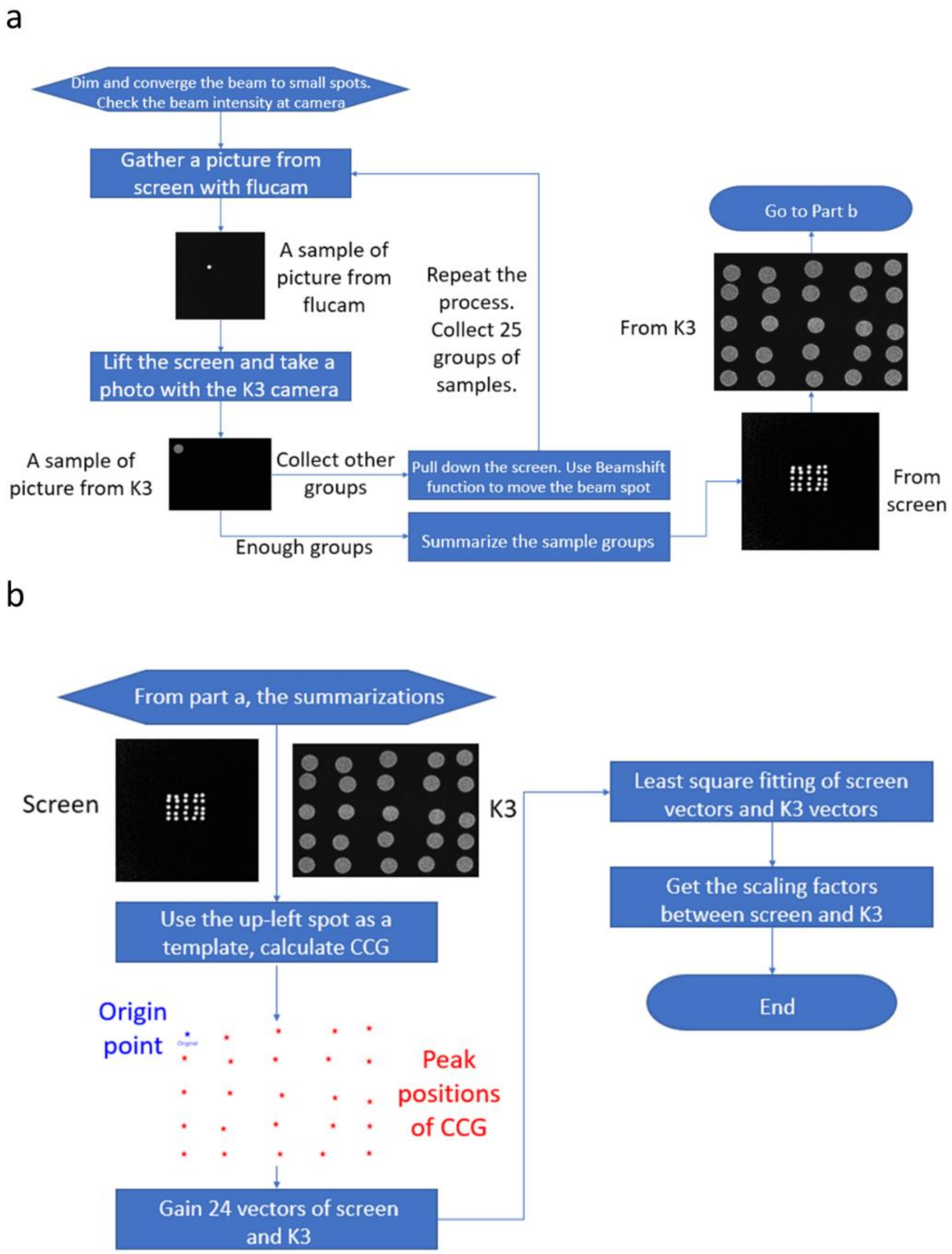

Supplementary Figure 2. Workflow of measuring scaling factors of K3.

a. The workflow chart for recording images of the same beam-spot on fluorescent screen camera and K3. b. The workflow chart of calculating the scaling factors of area between fluorescent screen and K3.

Supplementary Figure 3.

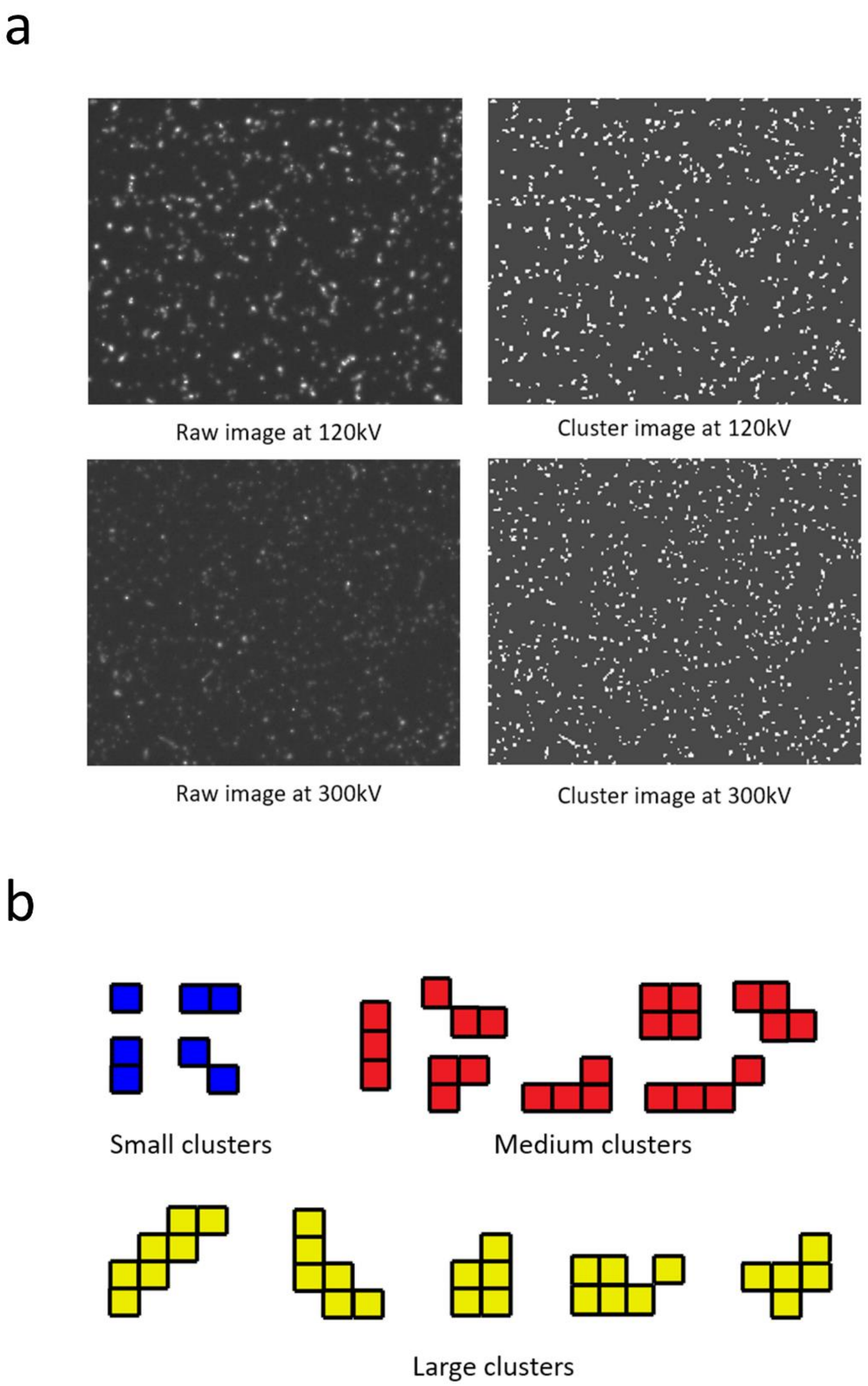

Supplementary Figure 3. Samples of raw images and cluster images of Falcon III.

a. Raw image and corresponding image of cluster. Left row, sample images of Falcon III camera in MFCDS mode (raw image). The display parameter in “v2” program of EMAN[1] package is B=-0.09, C=0.6. Right row, sample images of clusters from left images. All pixels in a cluster have value 1, the background being zero. b. Examples of shape of small in blue, medium in red and large clusters in yellow.

Supplementary Figure 4.

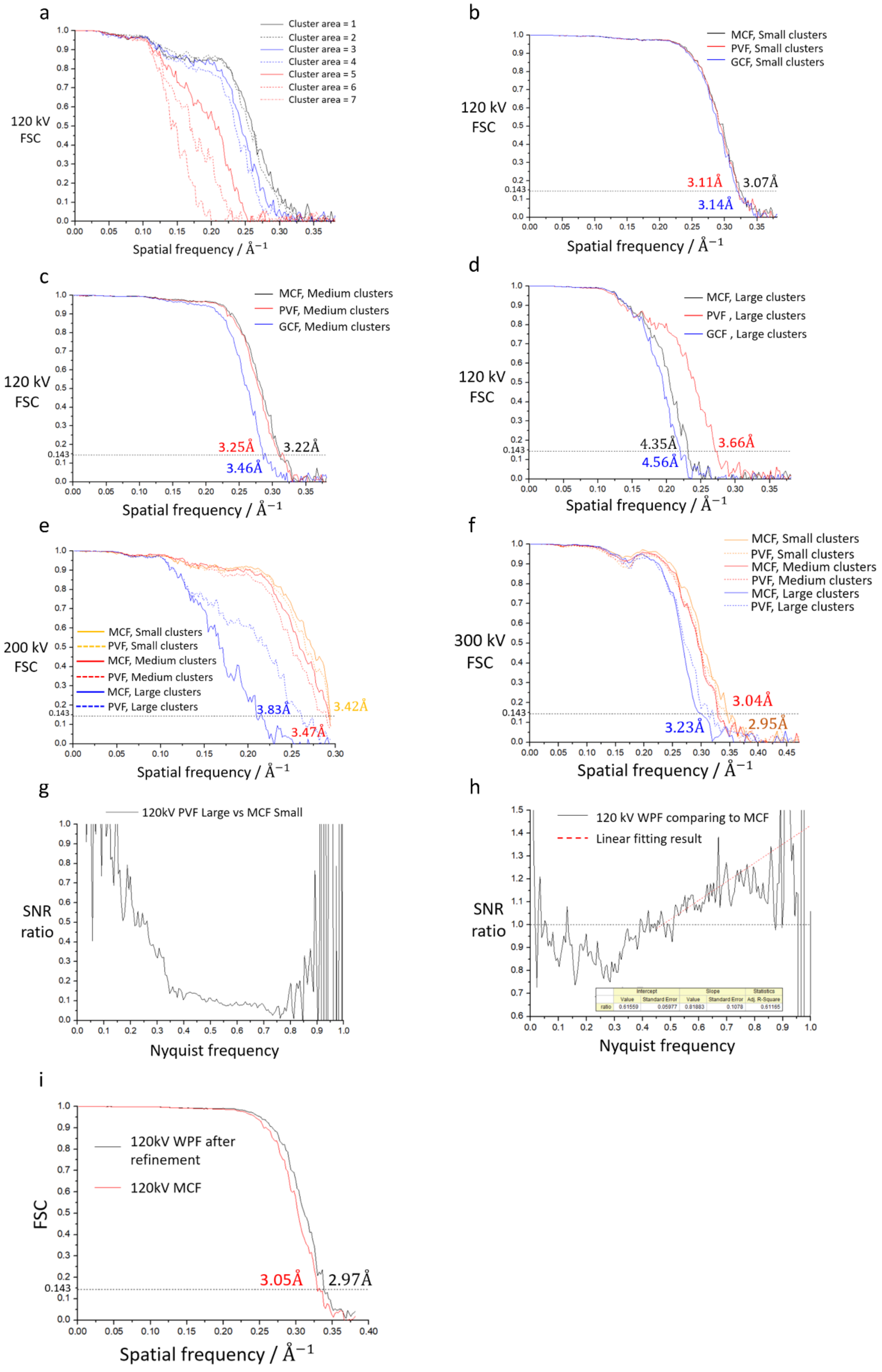

Supplementary Figure 4. Performances of counting algorithm that filtering cluster into single point.

a. Fourier shell coefficient (FSC) curves of clusters by their sizes at 120 kV. Clusters from “small/medium/large-sized” are displayed in the same colour of black, blue and red. MCF algorithm was used to filter each cluster, and alignment was the same. Each reconstruction contains roughly the same number of clusters. b. Fourier shell coefficient (FSC) curves of “small-sized” clusters in Coxsackievirus A10 dataset at 120 kV with MCF, PVF and GCF counting algorithm. Each pair of reconstructions holds roughly the same number of clusters (~81.4% of all “small-sized” clusters). The resolution of each counting algorithm mark on the graph. c. FSC curves of “medium-sized” clusters in Coxsackievirus A10 dataset at 120 kV with MCF, PVF and GCF counting algorithm. Each pair of reconstructions holds roughly the same number of clusters (~71.3% of all “medium-sized” clusters). The resolution of each counting algorithm mark on the graph. d. FSC curves of “large-sized” clusters in Coxsackievirus A10 dataset at 120 kV with MCF, PVF and GCF counting algorithm. Each pair of reconstructions holds roughly the same number of clusters (100% of all “large-sized” clusters). The resolution of each counting algorithm mark on the graph. e. FSC curves of 3 types of clusters in Coxsackievirus A10 dataset at 200kV with MCF and PVF counting algorithm. Each pair of reconstructions holds roughly the same number of clusters (18.3% of “small-sized” clusters, 51.2% of “medium-sized” clusters and all “large-sized” clusters). The resolution of PVF counting mark on the graph. f. FSC curves of 3 types of clusters in apo-ferritin dataset at 300 kV with MCF

and PVF counting algorithm. Each pair of reconstructions holds roughly the same number of clusters (12.5% of “small-sized” clusters, 24.8% of “medium-sized” clusters and all “large-sized” clusters). The resolution of PVF counting mark on the graph. g. SNR ratio between “large-sized” clusters of PVF and “small-sized” clusters of MCF at 120 kV. The dataset is Coxsackievirus A10. No alignment was changed when comparing. h. SNR ratio between WPF and MCF. The linear fitting interval is 0.45~0.65 Nyquist frequency. No alignment was changed when comparing. i. FSC curves of WPF after refinement versus MCF at 120 kV. The dataset is Coxsackievirus A10.

Supplementary Figure 5.

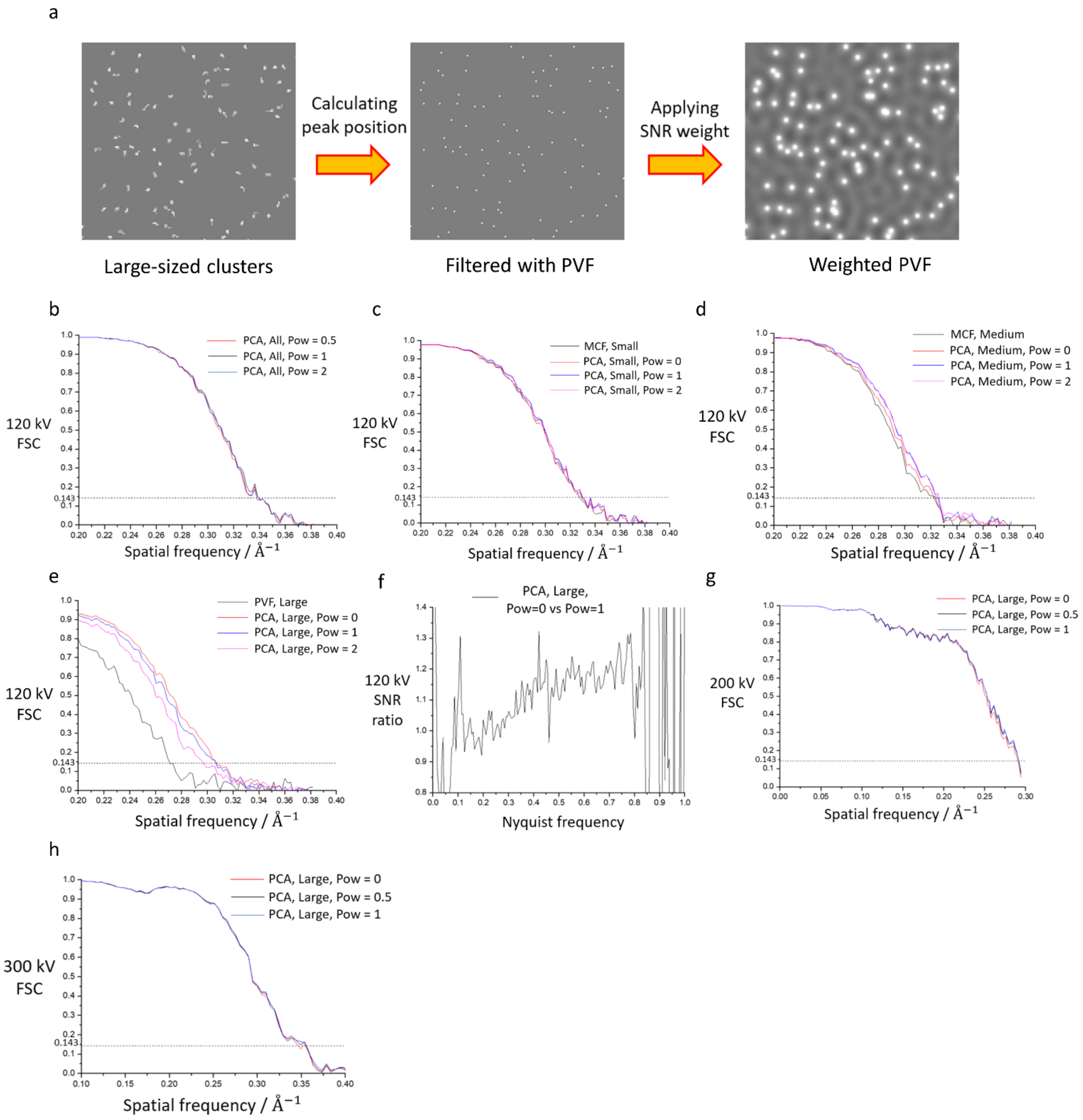

Supplementary Figure 5. Performances of PCA.

a. Shape of SNR-weighted PVF for “large-sized” clusters. The SNR-weight was applied in Fourier space, therefore some negative value was on “Weighted PVF” graph which shown in real space. b. FSC curves of “*pow*” parameter testing on all clusters at 120 kV. Every cluster was used for reconstruction. c. FSC curves of “*pow*” parameter testing on “small-sized” clusters at 120 kV. Every cluster was used for reconstruction. d. FSC curves of “*pow*” parameter testing on “medium-sized” clusters at 120 kV. Every cluster was used for reconstruction. e. FSC curves of “*pow*” parameter testing on “large-sized” clusters at 120 kV. Every cluster was used for reconstruction. f. Comparison of SNR for “large-sized” cluster between “*pow*=0” and “1”. The dataset is Coxsackievirus. g. FSC curves of “*pow*” parameter testing on “large-sized” clusters at 200kV. Every cluster was used for reconstruction. The dataset is Coxsackievirus A10. h. FSC curves of “*pow*” parameter testing on “large-sized” clusters at 300 kV. Every cluster was used for reconstruction. The dataset is apo-ferritin.

Supplementary Figure 6.

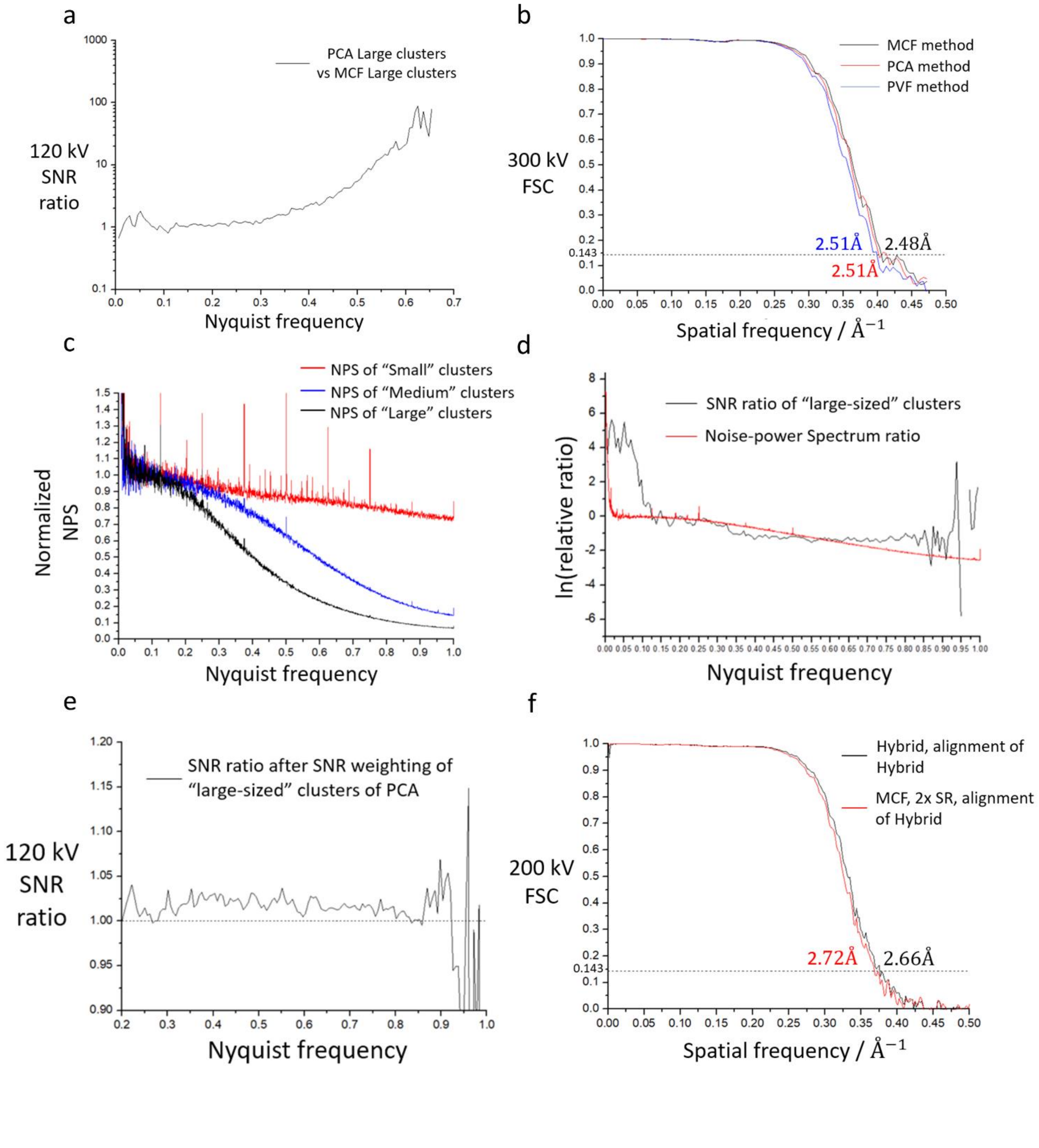

Supplementary Figure 6. SNR weight of PCA and performance of Hybrid counting algorithm.

a. Comparison of SNR at 120 kV of “large-sized” clusters between PCA and MCF. b. FSC curves of PCA, PVF and MCF counting algorithm in combined images of apo-ferritin dataset at 300 kV. Alignment file from MCF method was used. The resolution of reconstructions marks on the graph. c. Normalized noise-power-spectrum (NPS) curves of “small/medium/large-sized” clusters from PCA method. Three curves were normalized so that their intensity at near zero frequency is 1. The Vertical artefacts on each curve come from the bad channels on Falcon III camera we used. d. SNR and NPS ratio between “large” and “small-sized” clusters in logarithm scale at 120 kV. PCA was used. e. SNR ratio after applying SNR weight to “large-sized” clusters counted by PCA at 120 kV. f. FSC curves of 2x2 super-resolution Hybrid and MCF algorithm in Coxsackievirus A10 dataset at 200kV. Alignment of Hybrid was used. The resolution of reconstructions marks on the graph.

Supplementary Figure 7.

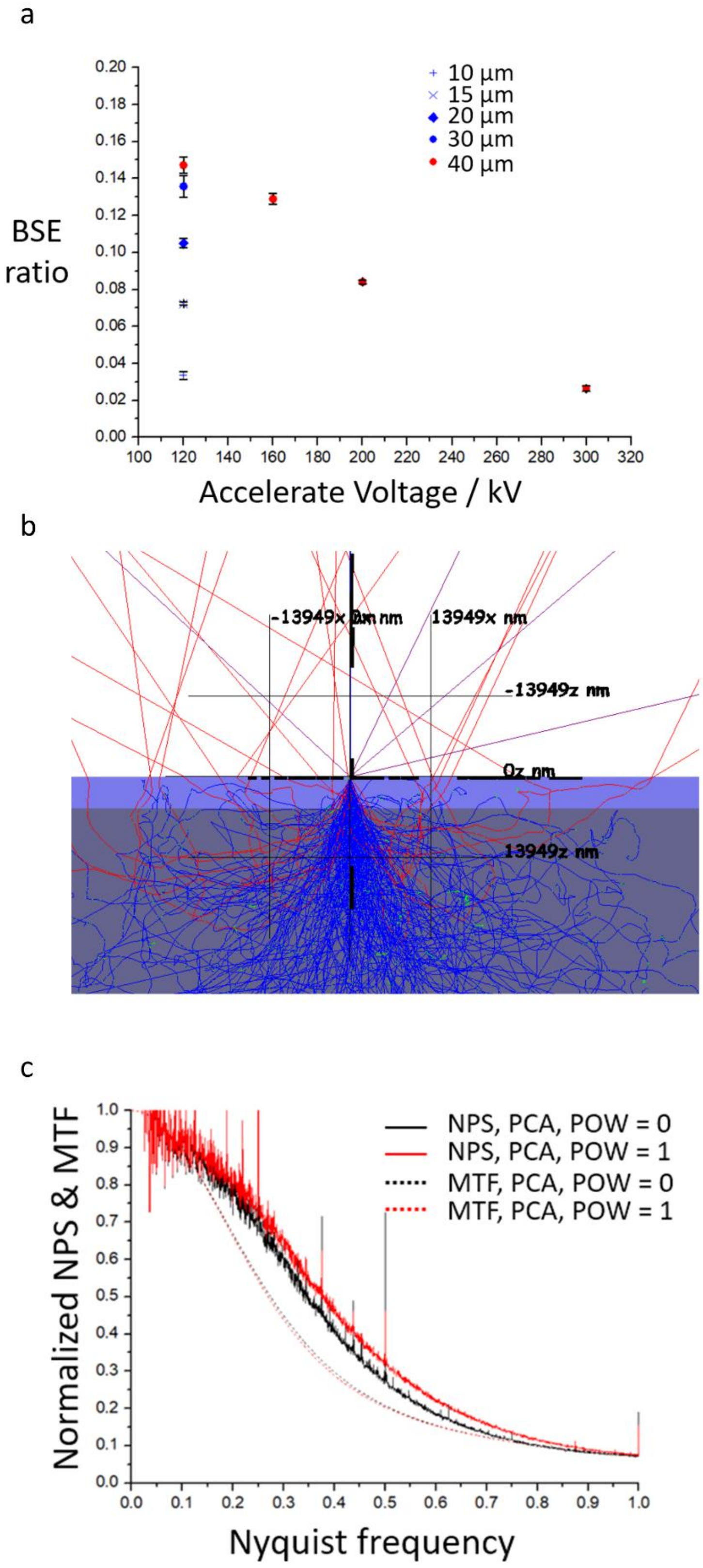

Supplementary Figure 7. Monte-Carlo simulations of BSEs.

a. Back-scattering-electron ratio by Monte-Carlo simulations using CASINO[2] package. Error bars represent 1 standard deviation. b. An example of Monte-Carlo simulations at 120 kV. c. Normalized NPS and modulation transfer function (MTF) of PCA method at 120 kV.

Supplementary Figure 8.

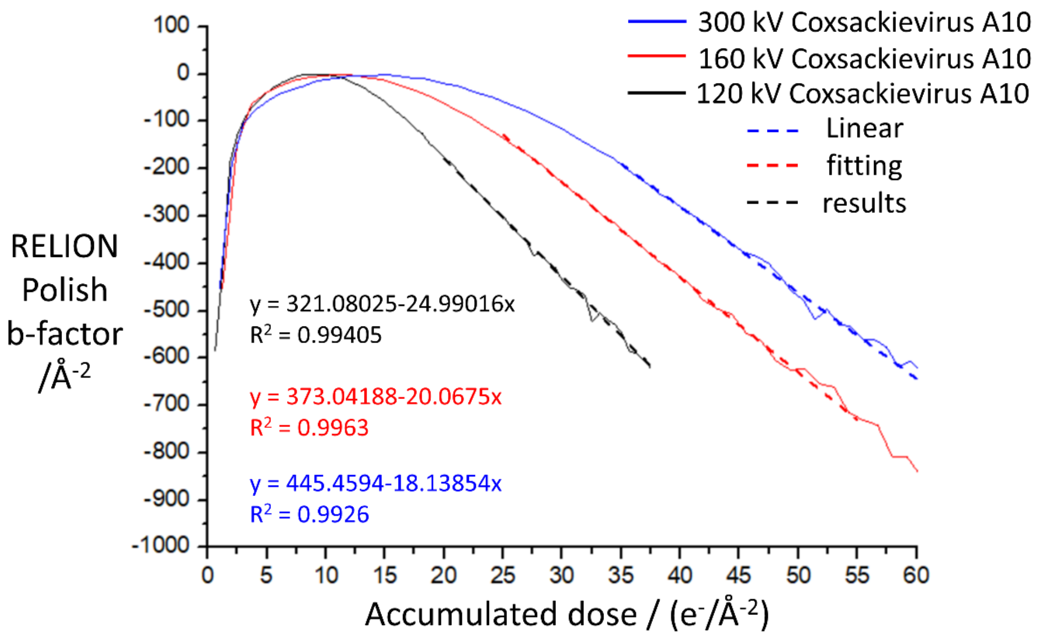

Supplementary Figure 8. B-factor plots from RELION polish at 300/160/120 kV for critical dose measurement.

The B-factor plots from RELION polish at 300/160/120 kV were shown in black, blue and red, respectively. Coloured dash lines show the linear fitting result at three voltages. At 120, 160, 300 kV, fitting range was from 20 to 38, 25 to 55 and 35 to 60 e-/Å^{-2}. The fitting functions and values of R-square marked on the figure.

Supplementary Figure 9.

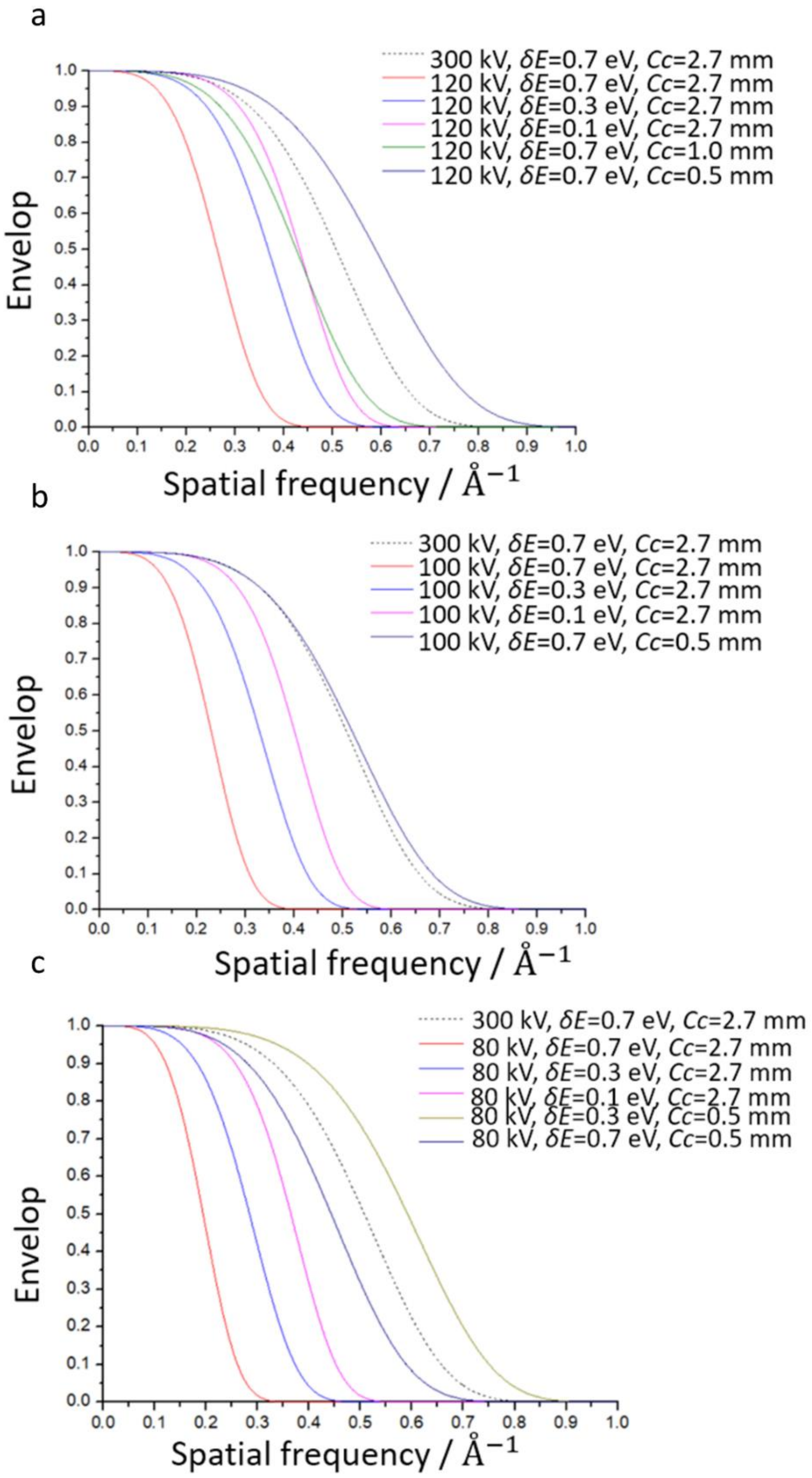

Supplementary Figure 9. Envelop by chromatic aberration.

a. Envelop by chromatic aberration at 120 kV. b. Envelop at 100 kV. c. Envelop at 80 kV. The δE and Cc we used were marked on figure. See Supplementary Note 9 for detail.

Supplementary Figure 10.

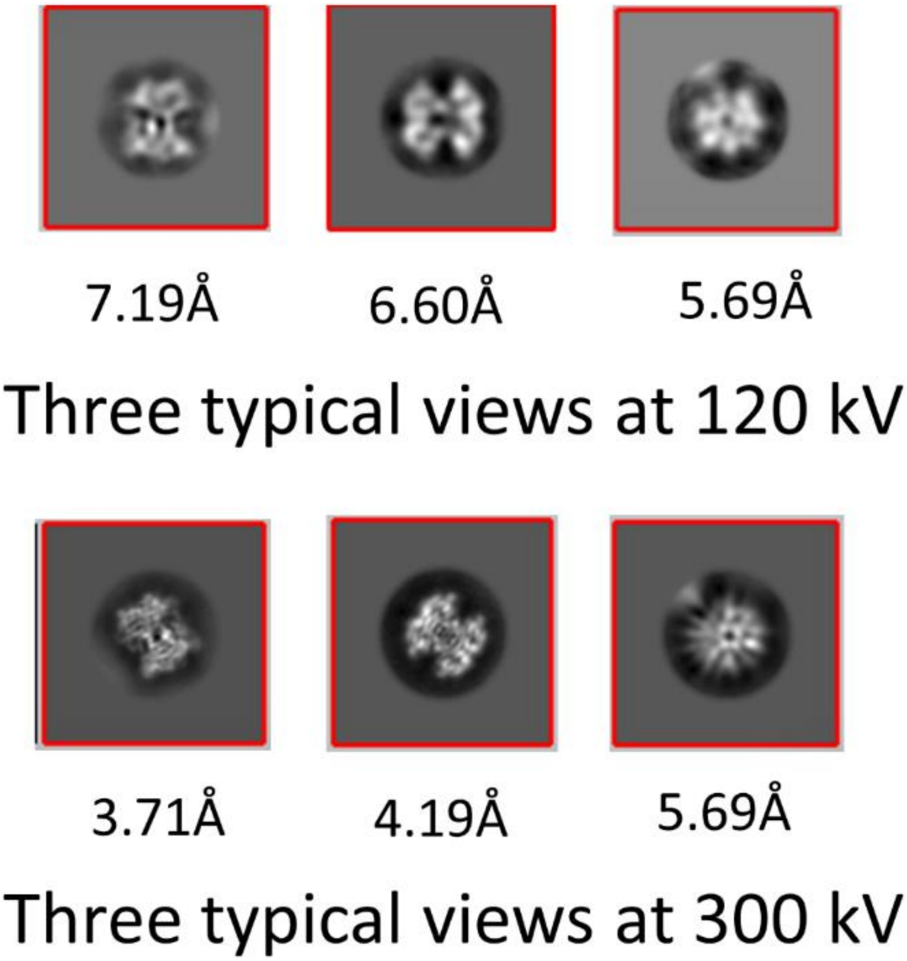

Supplementary Figure 10. 2D-classification results of apo-streptavidin at 120/300 kV.

Total number of ~1,250,000 particles at 300 kV with pixel size of 1.66 Å (Bin-2) and ~1,410,000 particles at 120 kV with pixel size of 1.72 Å (Bin-2) was used. 25 iterations and 100 classes were used during classification by RELION3. Data was collected by Falcon III detector with Hybrid counting at 120 kV and K3 detector with non-CDS bin-0.5 mode at 300 kV. Three typical views were selected from the 2D class averages and the corresponding resolution reported by RELION program was listed below the view.

Supplementary Figure 11.

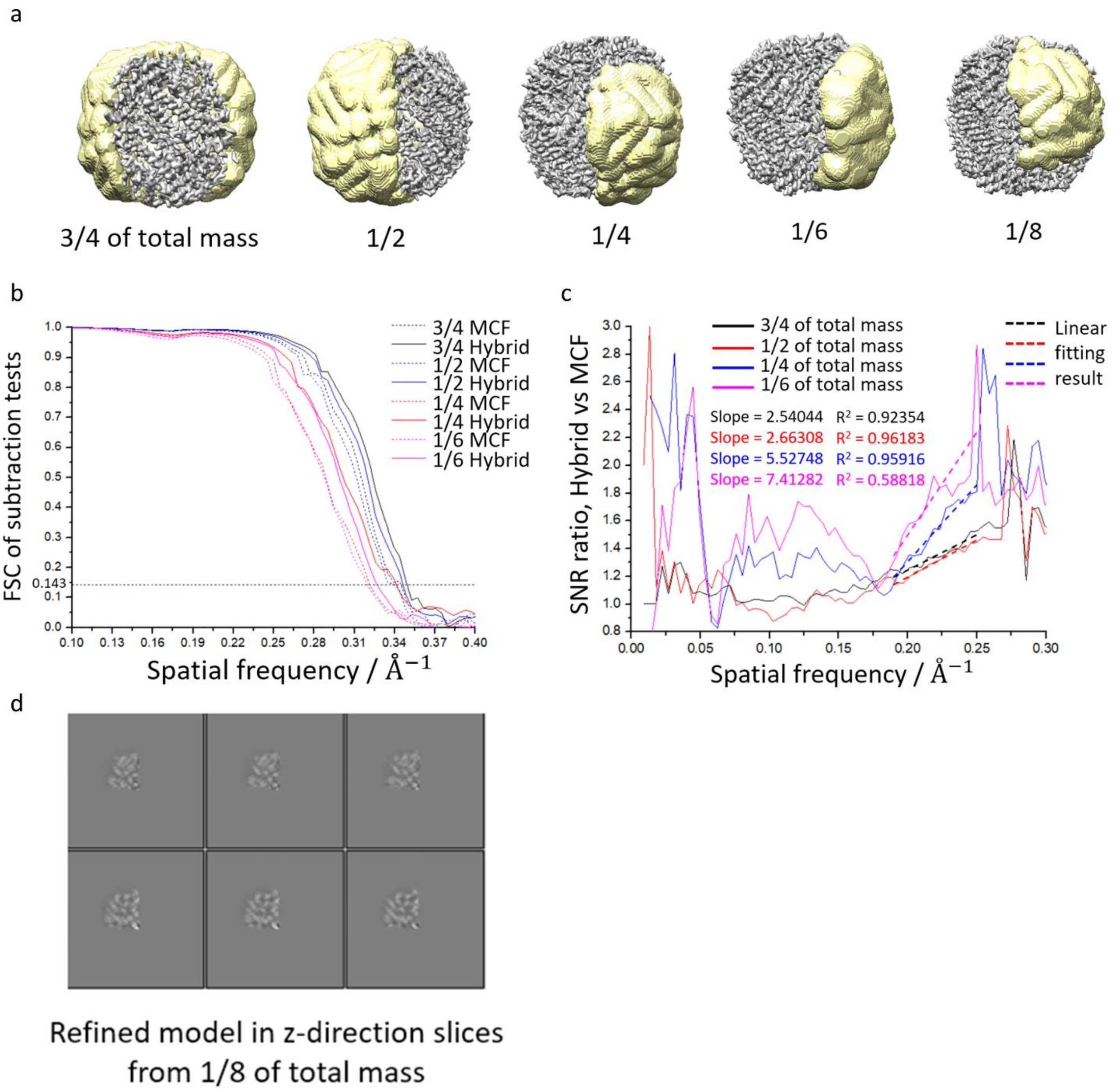

Supplementary Figure 11. Subtraction test of apo-ferritin at 120 kV.

a. Masks used for refinement. The densities outside the mask were subtracted from the 2D images. The remaining mass ratio in mask is marked under the mask. 340 kD (3/4), 225 kD (1/2), 113 kD (1/4), 75 kD (1/6) and 56 kD (1/8 of total mass) is included in each mask, respectively. b. FSC plots of the subtraction tests c. The SNR ratio between Hybrid and MCF. Linear fitting range is 0.18 to 0.25$Å^{-1}$ to prevent from dips on FSC curves. d. Some slices in z-direction of 1/8 mask. See Supplementary Note 10 for detail.

Supplementary Figure 12.

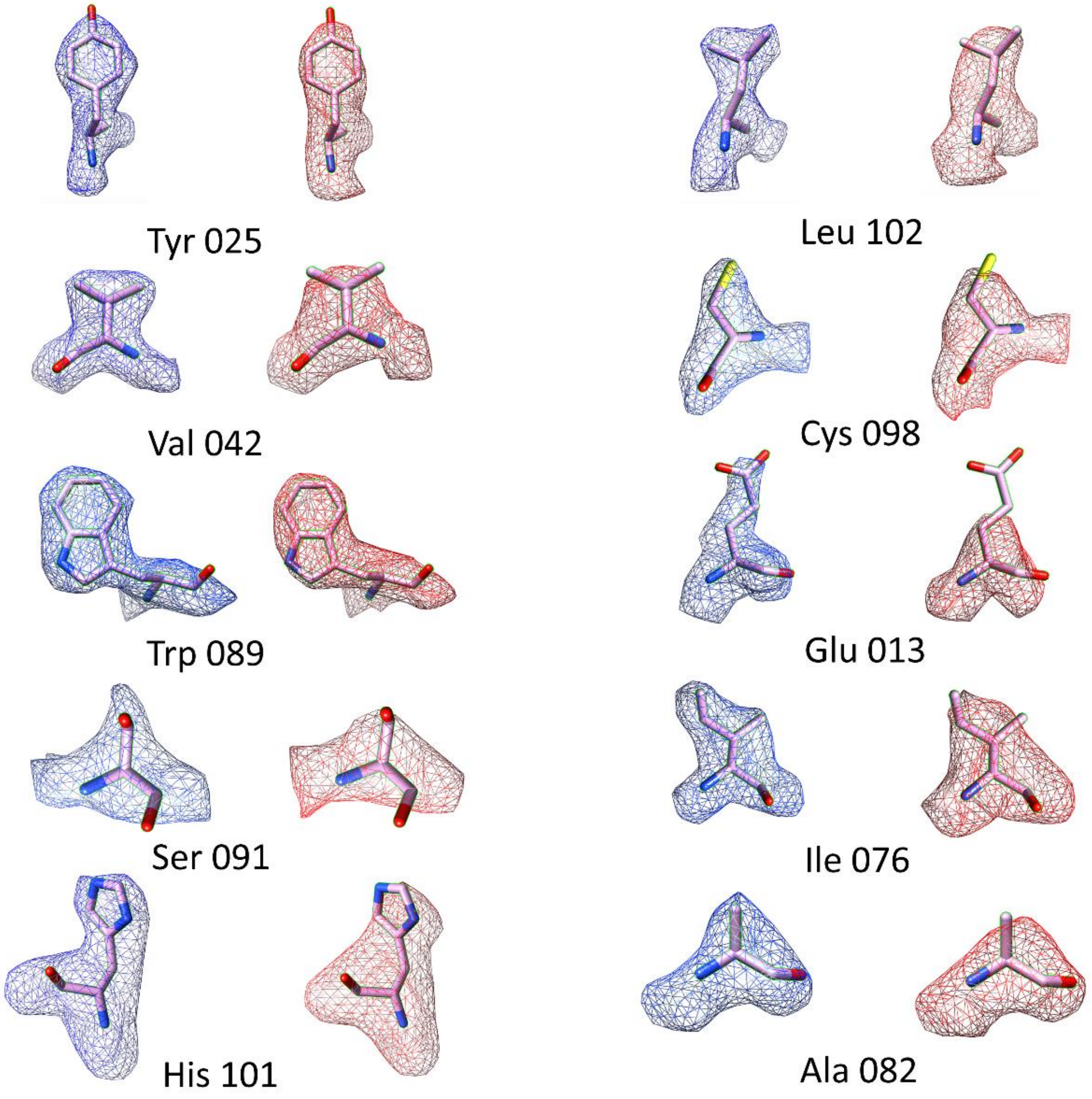

Supplementary Figure 12. Samples of side-chain on dataset of apo-ferritin at 120 kV with Hybrid and MCF method.

The densities in blue or red mesh represent the map from Hybrid or MCF method, respectively. Final resolution of Hybrid and MCF method is 2.87 Å and 3.39 Å, respectively. The name and number of residues marked below each group.

Supplementary Table 1.

Voltage /kV	X-axis scaling factor	Y-axis scaling factor	Scaling factor of area	Linear fitting slope between readouts and SC	R-Square	Counting scaling factor
300	20.16805	19.94657	1.00	6.85625	0.999974	1.000
160	20.56655	20.69969	1.05826	9.09105	0.99967	1.403
120	20.13949	19.88695	0.9956	10.32983	0.999994	1.500

Supplementary Table 1. The counting scaling factor at 300/160/120 kV.

The reference is 300 kV. X/Y-axis scaling factors were measured according to Supplementary Figure 2. Linear fitting slopes were the fitting results from Fig 1a.

Supplementary Note 1. Measuring the intensity homogeneity of beam spot.

The homogeneity of beam spot was tested within large illuminated area (IA) and small IA. When setting IA, the beam spot was expanded large enough while keeping parallel illumination. Beam-shift was used so that the K3 camera stayed at 5 positions of the beam spot, as shown on Supplementary Figure 1b and the exact position shown on Supplementary Figure 1c. We then measured the readout of K3 camera 5 times at each position by DigitalMicrograph software (Gatan Inc.), the exposure time being 0.3s. The standard deviations of the beam at 120 kV are 0.66% and 0.69% for small IA and large IA, respectively.

Supplementary Note 2. Multiple-point-fitting measuring the relative area occupation of K3 camera to fluorescent screen.

Because of the convergence of beam, the occupied area of K3 at fluorescent screen might be changed at different voltage. Data was acquired at a magnification of 22,500x. Spot sizes from 8 to 11 were used to prevent beam from damaging K3 after shrinking into a small spot. Then beam-shift was used, which did not change the beam shape and intensity, to move the spot at certain positions. At each position, TEM Imaging & Analysis (TIA, Thermo Fisher) software was used to take a shot from fluorescent screen camera (flucam), and DigitalMicrograph was used to take another shot from K3. Total of 25 shots were taken at each voltage, and peak value of cross-correlation-graph was used to find the exact position of beam spot on each shot. As a result, two groups consisting of 24 vectors were calculated at each voltage. The least-squares-fitting method was then used to fit two groups of vectors and the relative area occupations were calculated. At 120 kV, the area occupation was 99.56% comparing to 300 kV, and at 160 kV the result was 105.83%. The charts of workflow are shown on Supplementary Figure 2a and 2b.

Supplementary Note 3. Measuring K3 readouts at three voltages.

Using the fluorescent screen current as a reference, the readouts of K3 was measured at different voltages. In each measurement, the beam was adjusted so that it completely covered the fluorescent screen and the strong Fresnel rings was outside of the screen while keeping parallel illumination. The SC value was noted, and the dose rate of K3 was measured by DigitalMicrograph with each exposure time of 0.2~0.3s, 5 times each. The results were linear-fitted and shown on Fig. 1a and Supplementary Table 1. According to the conservation of the number of electrons, the relationship between SC and read-out of K3 can be calculated by this Supplementary Eq.1:

$$SC \cdot Si = EPS \cdot pixels \tag{1}$$

Where *Si* is the related area occupation of K3 at certain voltage, $pixels$ is the pixel number of K3, being a constant, and *EPS* is the real dose rate of K3. As a result, the counting scaling factor (CSF) can be calculated and shown on Supplementary Table 1. The CSF and readouts of DigitalMicrograph were used to determine the relative dose rate at three voltages.

Supplementary Note 4. Measuring the relative critical dose at three voltages.

A modification of relion_motion_refine[3,4] program was used to generate particle-images from each frame while keeping per-particle tracks and without frequency weighting[5]. These particles in the same frame series were reconstructed by relion_reconstruct program and FSC curve of each reconstruction was calculated by relion_postprocess program and converted to SNR curve by Eq.2[6]. A script was used to combine the SNR curve into "plots of ln(SNR) vs accumulated exposure[7]". The linear-fitting of the plots started from 15th frame to 30th frame and critical doses were calculated by the fitting results. After obtained the slopes, data in frequency between 10 Å and 5 Å was used to calculate the relative ratio between each voltage.

Supplementary Note 5. Determination of clusters on Falcon III.

The counting programs we developed based on EMAN[1] input/output libs read the raw images from MFCDS mode on Falcon III. MFCDS mode applies pre-EC gain of Falcon III images. Images were firstly thresholding by parameter "-m" to drop the background noise, generating image "A0". A 5-tap Laplacian-of-Gaussian kernel was convoluted to A0 and then thresholding by parameter "-x" into a binary map (0 or 1, 0 is the background). Connected-component labelling algorithm[8] was used and any 8 nearest non-Zero points beside a non-Zero point was considered belonging to the same cluster. Optimization of "-m" and "-x" was done by brute force search of total number of clusters and by eye-witness. The sets of parameters we used were "-m 45 -x 35", "-m 30 -x 30" and "-m 30 -x 25" for 120 kV, 200 kV and 300 kV, respectively.

Supplementary Note 6. Filtering clusters

5 filters were used for pre-determined clusters. Mass-Centre (MCF) filter follows Supplementary Eq.2. When generating image stacks, Supplementary Eq.3 is used. “N=2”, meaning 2x2 super-resolution, is used for all images stacks generated by MCF and each MCF point has value of 1.

$$\overrightarrow{MCF(x,y)} = \frac{\sum \overrightarrow{(x,y)} \cdot value(x,y)}{\sum value(x,y)} \tag{2}$$

$$\overrightarrow{Image(x,y)} = round[N \cdot \overrightarrow{MCF(x,y)}] \tag{3}$$

Peak-value filter (PVF) follows Supplementary Eq.4, which means the max-value position is chosen to represent the incident position of electron. Each PVF has value of 1.

$$\overrightarrow{PVF(x,y)} = \max\,[\overrightarrow{value(x,y)}] \tag{4}$$

Geometric-Centre filter (GCF) follows Supplementary Eq.5. It neglected the value of pixels. Although GCF can also have super-resolution mode, it had not been implemented during our experiments.

$$\overrightarrow{GCF(x,y)} = \frac{\sum \overrightarrow{(x,y)}}{n} \tag{5}$$

PCA filter follows Supplementary Eq.6. Unlike 3 other filters that transform a cluster into a single point, it keeps the value and shape of the cluster. The *pow* parameter is an adjustable power of $value(x,y)$.

$$\{PCA(x,y,pow)\} = \frac{\{value(x,y)^{pow}\}}{\sum value(x,y)^{pow}} \tag{6}$$

Hybrid filter combines MCF and PCA. It uses MCF for small/medium clusters and PCA (x, y,0) for large-size clusters. In non-super-resolution mode, MCF with *N=2*

was used then twice binned in Fourier space. In NxN super-resolution mode, MCF with *N*=N was used, in the meantime images from PCA (x, y,0) are upscaled by a bicubic scaling function (https://www.paulinternet.nl/?page=bicubic).

Supplementary Note 7. SNR comparison on electron counting filters.

The same modified relion_motion_refine motioned above was used to produce particle images of each filter, except that the frequency weight from Bayesian Polish was kept. The SNR ratio between each filter was not been affected by applying the same frequency weight. On each filter, the two halves of particles were reconstructed and post-processed by relion_reconstruct and relion_postprocess, where the same mask was applied. The corresponding FSC curves were converted to SNR curves and ratio of SNR can be compared by dividing them.

Supplementary Note 8. Applying SNR weighting to images of "large-sized" clusters.

Suppose this scenario: Two micrograph F1 and F2 with known SNR_1 and SNR_2. Maximize SNR in Supplementary Eq.7 with weighting function *w* by ignoring the $N_1 \cdot N_2$ term:

$$\mathrm{SNR} = \left(\frac{S_1 + wS_2}{N_1 + wN_2}\right)^2 \sim \frac{(S_1 + wS_2)^2}{N_1^2 + w^2 N_2^2} \tag{7}$$

Where S_1, S_2 is signal of F1, F2 and N_1, N_2 is the noise. We use

$$\frac{\partial SNR}{\partial w} = 0 \tag{8}$$

then we have

$$2(S_1 + wS_2)S_2(N_1^2 + w^2 N_2^2) = (S_1 + wS_2)^2 2wN_2^2 \tag{9}$$

and

$$w = \frac{S_2 N_1^2}{S_1 N_2^2} = \sqrt{\frac{SNR_2}{SNR_1} \cdot \frac{N_1^2}{N_2^2}} \tag{10}$$

where

$$SNR_{1\backslash 2} = \left(\frac{S_{1\backslash 2}}{N_{1\backslash 2}}\right)^2 \tag{11}$$

The N_1^2, N_2^2 term of Supplementary Eq.11 can be replaced by NPS.

$$w = \sqrt{\frac{SNR_2}{SNR_1}} \cdot \sqrt{\frac{NPS_1}{NPS_2}} \tag{12}$$

Where NPS_1, NPS_2 is the Noise-Power-Spectrum of F1 and F2. The NPS can be calculated by Fourier transformation of image without any sample grid.

Supplementary Note 9. Calculating envelop function of chromatic aberration and depressed FSC.

The parameters we used for Eq.5 comes from Nakane et al.[9],

where $\frac{\delta(V)}{V} = 2 \cdot 10^{-8}$, $\frac{\delta(I)}{I} = 1 \cdot 10^{-7}$ and $\delta(E) = 0.7eV$ for XFEG. Since the envelop of chromatic aberration is a part of the amplitude of CTF, the effect on SNR should be proportional to its square. As a result, the FSC we predicted for low-voltage conditions can be represented by Supplementary Eq.13:

$$\mathrm{FSC}_{0.143}^{pred}(V,\omega) = \frac{\left(\frac{\mathrm{FSC}_{0.143}(\omega)}{1-\mathrm{FSC}_{0.143}(\omega)}\right)\cdot\left(\frac{ENV_{CC}^{2}(V,\omega)}{ENV_{CC}^{2}(\omega)}\right)}{1+\left(\frac{\mathrm{FSC}_{0.143}(\omega)}{1-\mathrm{FSC}_{0.143}(\omega)}\right)\cdot\left(\frac{ENV_{CC}^{2}(V,\omega)}{ENV_{CC}^{2}(\omega)}\right)} \tag{13}$$

Where the $\mathrm{FSC}_{0.143}(\omega)$ and $ENV_{CC}^{2}(\omega)$ is the FSC curve and envelop curve by chromatic aberration at 300 kV, $ENV_{CC}^{2}(V,\omega)$ is the envelop curve by chromatic aberration at lower voltage, and $\mathrm{FSC}_{0.143}^{pred}$ is the predicted FSC at lower voltage *V*.

Supplementary Note 10. Subtraction test of apo-ferritin at 120 kV.

The star-file of Hybrid counting method after refinement was symmetry-expanded from O-symmetry to C1 by relion_particle_symmetry_expand. We then used relion_particle_subtraction program to subtract the expanded star-file with masks. Each mask contains 3/4 to 1/8 of total mass and low-passed to 15 Å (Supplementary Figure 11 a). "--ignore_class --data expanded_data.star" was used for subtraction to force subtracting to given expanded star-file. After subtracting Hybrid images, particle images from MCF replaced Hybrid images and the same processes were done one again. Since the subtraction is not perfect and many positive or negative features remained, new masks low-passed to 15 Å were used during Auto-refinement after iteration 3. The refinements were local, starting with angle steps of 1.8 degree. After refinement, SNR was calculated and compared followed latter part of Supplementary Note 7. We found the refined result of 1/8 (56 kD) is dramatically worse, shown on Supplementary Figure 11c, thus we excluded this result.

Supplementary References

1. Ludtke, S. J., Baldwin, P. R. & Chiu, W. EMAN: Semiautomated software for high-resolution single-particle reconstructions. *J. Struct. Biol.* **128**, 82–97 (1999).
2. Hovington, P., Drouin, D. & Gauvin, R. CASINO: A new Monte Carlo code in C language for electron beam interaction - Part I: Description of the program. *Scanning* **19**, 1–14 (1997).
3. Zivanov, J. *et al.* New tools for automated high-resolution cryo-EM structure determination in RELION-3. *Elife* **7**, 1–22 (2018).
4. Zivanov, J., Nakane, T. & Scheres, S. H. W. A Bayesian approach to beam-induced motion correction in cryo-EM single-particle analysis. *IUCrJ* **6**, 5–17 (2019).
5. Grant, T. & Grigorieff, N. Measuring the optimal exposure for single particle cryo-EM using a 2.6 Å reconstruction of rotavirus VP6. *Elife* **4**, 1–19 (2015).
6. Grigorieff, N. Resolution measurement structures derived from single particles. *Acta Crystallogr. Sect. D Biol. Crystallogr.* **56**, 1270–1277 (2000).
7. Stagg, S. M., Noble, A. J., Spilman, M. & Chapman, M. S. ResLog plots as an empirical metric of the quality of cryo-EM reconstructions. *J. Struct. Biol.* **185**, 418–426 (2014).
8. Vincent, L., Vincent, L. & Soille, P. Watersheds in Digital Spaces: An Efficient Algorithm Based on Immersion Simulations. *IEEE Trans. Pattern Anal. Mach. Intell.* **13**, 583–598 (1991).

9. Nakane, T. *et al.* Single-particle cryo-EM at atomic resolution. *Nature* **587**, 152–156 (2020).
