# Supplementary material for: An electron counting algorithm improves imaging of proteins with low-acceleration-voltage cryo-electron microscope
Source: Commun Biol. 2022 Apr 6;5:321. doi: 10.1038/s42003-022-03284-1 (PMC8987035; doi:10.1038/s42003-022-03284-1)
Supplement: Supplementary file 3 — Description of Additional Supplementary Files [file 42003_2022_3284_MOESM3_ESM.pdf]

Description of Additional Supplementary Files

File name: Supplementary Data 1.
Description: Source data of graphs and charts presented in the main figures.
